# Views of Dutch general practitioners about premenstrual symptoms: A qualitative interview study

**DOI:** 10.1080/13814788.2021.1889505

**Published:** 2021-03-17

**Authors:** Marijke S. Labots-Vogelesang, Doreth A. M. Teunissen, Vivianne Kranenburg, Antoine L. M. Lagro-Janssen

**Affiliations:** Gender and Women’s Health Unit, Department of Primary and Community Care, Radboud University Medical Centre, Nijmegen, The Netherlands

**Keywords:** Premenstrual syndrome, PMS, PMS aetiology, general practitioner, qualitative interview

## Abstract

**Background:**

General practitioners (GPs) encounter women suffering from premenstrual symptoms. Often women with premenstrual problems experience little understanding from GPs. Views of GPs will influence their approach to these women and their care. Insight into these views is lacking but could help in designing educational programmes for GPs.

**Objectives:**

To explore the views of Dutch GPs towards aetiology, diagnostic process, and preferred treatment of premenstrual symptoms.

**Methods:**

In 2017, we conducted a qualitative, semi-structured, interview survey among 27 GPs, varying in age, gender, and practice setting.

**Results:**

Important themes emerged from the interviews: ‘no need for a symptom diary,’ ‘PMS defined as illness’ exclusively in case of disruption of normal functioning, and ‘symptomatic treatment’ as preferred approach. Most GPs considered PMS to be a physiological phenomenon, with taking history as an adequate diagnostic tool. Almost all GPs regarded a normal cyclical hormonal cycle as causal; many also mentioned the combination with personal sensitivity. Some pointed to a dividing line between physiological condition and illness if women could not function normally in daily life. Lastly, the approach GPs preferred was focussing on relieving symptoms of individual patients. In addition to explaining the hormonal cycle and lifestyle advice, all GPs advocated oral contraceptives, and if necessary psychological support. GPs expressed negative feelings about prescribing antidepressants.

**Conclusion:**

GPs considered physiological changes and personal sensitivity as aetiological factors. We recommend more training to improve GPs knowledge and more insight into the burden of women with PMS. A symptom diary is an essential diagnostic tool for GPs.

KEY MESSAGES

- GPs consider PMS as a physiological hormonal condition, triggered by a personal sensitivity, not as an illness
- GPs mainly use history taking to diagnose PMS, without a symptom diary as diagnostic tool
- GPs prefer a symptom-oriented approach with explanation, lifestyle advice, contraceptive hormones and if necessary psychological support

## Introduction

Premenstrual syndrome (PMS) covers a broad spectrum of physiological and psychological symptoms that may disrupt women’s daily lives. Characteristic of PMS is the cyclic occurrence of symptoms in the luteal phase of the menstrual cycle [1]. There is a strict distinction between PMS and Premenstrual Dysphoric Disorder (PMDD), described in the DSM-5 as set out in Box 1. Compulsory diagnostic criteria for PMDD are that in most menstrual cycles during the past year, five (or more) of the symptoms described in Box 1 occurred during the final week before the onset of menses. These started to improve within a few days after the onset of the menses, and the symptoms were minimal or absent in the week post menses, regarding at least one of the emotional symptoms lability, irritability, depressed mood, anxiety and as physical symptoms painful breasts and bloating.

Box 1 Criteria for Premenstrual Dysphoric Disorder as defined in the DSM-5

- In most menstrual cycles during the past year, five (or more) of the following symptoms occurred during the final week before the onset of menses, started to improve within a few days after the onset of the menses, and were minimal or absent in the week post menses, with at least one of the symptoms being either (1), (2), (3) or (4):
  1. Marked affective lability (e.g. mood swings, feeling suddenly sad or tearful or increased sensitivity to rejection);
  2. Marked irritability or anger or increased interpersonal conflicts;
  3. Marked depressed mood, feelings of hopelessness, or self-deprecating thoughts;
  4. Marked anxiety, tension, feelings of being ‘keyed up’ or ‘on edge;’
  5. Decreased interest in usual activities (e.g. work, school, friends, hobbies);
  6. Subjective sense of difficulty in concentrating;
  7. Lethargy, easy fatiguability, or marked lack of energy;
  8. Marked change in appetite, overeating, or specific food cravings;
  9. Hypersomnia or insomnia.
  10. A subjective sense of being overwhelmed or out of control.
  11. Other physical symptoms, such as breast tenderness or swelling, joint or muscle pain, a sensation of ‘bloating,’ weight gain.
- The symptoms are associated with clinically significant distress or inference with work, school, usual social activities or relationships with others (e.g. avoidance of social activities, decreased productivity and efficiency at work or school).
- The disturbance is not merely an exacerbation of the symptoms of another disorder, such as major depressive disorder, panic disorder, dysthymic disorder, or a personality disorder (although it may co-occur with any of these disorders);
- Criteria A, B, and C must be confirmed by prospective daily ratings during at least two symptomatic cycles. (The diagnosis may be provisionally prior to this confirmation);
- The symptoms are not due to the direct physiological effects of a substance (e.g. drug of abuse, a medication, or other treatment) or a general medical condition (e.g. hyperthyroidism).
  © American Psychiatric Association. 2013. ISBN 978-0-89042-554-1

PMS, which is not an official DSM-5 diagnosis, requires only one clinically significant cyclical symptom across the cycle that can be physical or psychological. The severity of the complaints distinguishes between PMS and PMDD. There are several homonymous descriptions of PMS without a numerical criterion for complaints [2,3]. The most common symptoms of PMS are painful breasts, bloating and emotional symptoms of which irritability is the most characteristic phenomenon [4].

Prospective and retrospective European studies have shown that 4–8% of women of fertile age, with hormonal cycles, have moderate to severe symptoms [4,5]. The reported diversity in the prevalence of PMS in the literature may depend on beliefs about the syndrome and in particular on the definitions used [2–8]. A lower threshold value for complaints at PMS than at PMDD directly implies a higher prevalence than in case of the strict criteria according to the DSM-5.

The aetiology of PMS is an issue of ongoing research and debate, as we still do not fully understand who has it and what causes it. Currently, there is more clarity about the underlying pathophysiology of an abnormal sensitivity and excessive response to normal hormonal changes [9,10].

Hormonal changes disturb the neurotransmitter metabolism and thus affect the physiological stress response in women with PMS. This may result in more susceptibility to stress and more negative emotions [9,11–13]. Especially in relationships, stress and ways of coping with PMS can strongly influence women´s distress. This altered stress response pattern appears to be independent of the menstrual cycle [11–14]. Personal sensitivity, genetic and evolutionary factors can play a role in this [11,14,15].

Women suffering from PMS may experience a high symptom burden and serious impairment in daily life and will therefore seek help [5]. The psychic symptoms strongly interfere with their self-perception and feminine identity. GPs are the professionals most likely to be called upon to deal with women with PMS. Women often mention experiencing little acknowledgement and understanding from their GP when they consult with premenstrual symptoms and problems [personal communication]. To our knowledge, there is no consensus guideline for primary care about PMS and the perspectives and attitudes of GPs towards PMS have been scarcely investigated. The existing consensus developed by the International Society for Premenstrual Disorders is based on specialist research [16].

To improve the quality of care for women with PMS, we aimed to investigate the views of GPs about PMS, how GPs in daily practice diagnose complaints fitting PMS and how they prefer to address the problem. We formulated the following questions: how do Dutch GPs understand and explain the aetiology of PMS, how do they make a diagnosis and what do GPs think is the most appropriate treatment of PMS?

## Methods

### Study design

We performed a qualitative study based on individual in-depth semi-structured interviews.

For practical reasons, we preferred to conduct interviews above focus groups to reach GPs, willing to discuss PMS, from all over the country, with possible diversity in gender, age, practice and place of residence.

### Study population

We recruited GPs through work-related networks using a purposive sampling strategy aiming for sufficient variation in age, gender, characteristics of practice (solo, duo, healthcare centre, consultation days per week), practice population and settings (city: >200,000, suburban: 50,000–200,000, rural: <50,000 inhabitants and sparsely populated area) and location in the Netherlands. We invited participants by telephone or e-mail. GPs received an explanation about the research project by e-mail. The GPs filled out a questionnaire on the above mentioned background characteristics.

### Data collection

In early 2017 two experienced researchers from the Department of Primary and Community Care and Gender & Women’s Health (ML, VK) at Radboud University Medical Centre conducted in-depth, semi-structured interviews using an interview guide. The interview guide was compiled based on the literature and supervisors’ expert opinions (ML, TT, ALM) (Box 2).

Box 2 Interview topic guide

Views about PMS
   What is your opinion about the aetiology and the illness  concept of PMS?
  How do you diagnose PMS complaints and symptoms?
  Views about treatment of PMS
   What is your first option in the approach to PMS?
   Which treatment do you prefer for PMS? And why?
   Which treatment don´t you prefer? And why?
   What is your view on psychological treatment and on   medication as hormones and antidepressants?

We interviewed the participants by telephone for practical reasons and based on findings indicating that telephone interviews are as valuable as face-to-face interviews [17]. The participants were asked to give their written informed consent prior to the interviews. Two interviews were used as a pilot; these are not included in the study. Participants received no reward.

### Analysis

Interviews were recorded on audio equipment with the participants’ consent and were transcribed verbatim. Participants’ names were removed from the transcripts and only the researchers had access to the original interviews. Two researchers (ML, VK) independently analysed the transcripts using Atlas.ti, version 7.1.5 [18]. Coding was done following open, axial and selective coding. Two independent researchers read the transcripts and used open coding to conceptualise the data. The codes were compared and discussed after every five interviews to reach consensus. When open coding was completed, axial coding was started by reflecting on the codes and rewriting them in categories. Lastly, these categories were conducted in overarching themes [19]. The researchers discussed all findings with the supervising committee (TT, ALM) until they reached consensus. After 21 interviews no more new codes were found, and thus saturation was achieved. We provided figures to indicate whether the results had been obtained from few (1–3), some (4–8), many (9–18), or most (19–28) participants. COREQ criteria were applied for reporting qualitative research [20].

## Results

We invited 30 GPs for an interview. In total, 27 consented to participate, of whom were ten males and 17 females with a mean age of 58.8 years. After reaching saturation, we still took another six interviews due to logistical agreements. The sample was diverse regarding years of practising and other background features. An overview of the characteristics of the participants is presented in Table 1.

**Table 1.** Participants’ characteristics (*n* = 27).

Gender		
Female		17
Mean age yrs. (SD)		44.4 (10.5)
Male		10
Mean age yrs. (SD)		58.5 (11.8)
Age (years)	≤ 40	8
	41-55	8
	> 55	11
Years practicing	2-10	6
	11-20	6
	21-30	7
	31-41	6
Consultation days p/week	Part-time ≤ 2	4
	Part time (> 2-4)	16
	Full time	5
Setting general practice	Solo	9
	Duo	8
	Healthcare centre	10
Area of practice situation	City	2
	Suburban	15
	Rural	10
Characteristics of practice population	Elderly patients	6
	Mainly adolescents/ young families	6
	Religious (reformed)	1
	Migrants	3
	Deprived area	3

For all GPs, history taking was based on the characteristic premenstrual symptoms. The aetiology of PMS was not a topic of debate among most participants. They did not perform a further investigation into a possible cause of the complaints. Their views on aetiology are reflected in the quotations presented.

We subdivided the relevant views emerging from our analysis of the data into the following themes: no need for a symptom diary; disruption of normal functioning as definition of illness; and lastly a symptomatic treatment approach.

### No need for a symptom diary

All GPs diagnosed PMS based on history taking with the cyclically related symptoms as the key point.

*‘Mostly, it is anamnestically quite clear, for the women themselves.’ P17, female, 38yrs*

The GPs did not perform a further investigation into a possible cause of the complaints, as they considered diagnosing not helpful. They did so because the definitive cause of PMS was not an issue in most of the participants.

*‘Well, we don’t know so. Point.’ P11, male, 65yrs*

A few GPs did use a tool like a symptom diary or a questionnaire to confirm the diagnosis.

### Disruption of normal functioning as definition of illness

Many GPs assumed that PMS is most likely related to cyclical hormone changes only and is therefore purely physiological.

*‘I see it as a problem, as a situation belonging to the normal physiological functioning of the body. There are women with dysmenorrhea, is that a problem? Yes, for women it may be a problem, but is it a medical problem? No, not really, but it can still bother them. […] It’s physiological, not an illness.’ P7, male, 58yrs*

Many GPs added that contextual factors as well as the patient´s suffering and coping strategies, contribute to being prone to premenstrual complaints.

*‘But I also think it has to do with environmental factors, psychological well-being, and balancing between suffering and coping strategies of patients.’ P8, female, 46yrs*

A few GPs put the symptoms under the umbrella of ‘medically unexplained symptoms’ (MUS). They seemed to regard PMS as a fashion trend that comes and goes like a wave movement.

*‘Anything that causes unwellness affects people who are sensitive to MUS (…).’ P15, male, 57yrs*

GPs also described patients’ individual sensitivity and mental well-being as contributing to PMS, as if passing a certain level.

*‘Well, […] people all have their own sensitivities and you have to pass a sort of threshold for the symptoms to appear. And I think those thresholds differ between people.’ P32, male, 54yrs*

Some GPs assumed the possibility of a genetic predisposition to develop PMS. A few other GPs mentioned that PMS is not purely physiological but an abnormal serotonin metabolism as potentially contributing to changes in serotonin activity related to mood state and developing PMS.

GPs considered PMS as an illness, only in case severe symptoms forced women to seek help. The dividing line, they found, was the point at which PMS disrupted women's daily life, when they suffered from anger, felt anxious, guilty or gloomy, in such a debilitating way, that they could no longer function normally.

*‘There are, of course also different graduations.[…] And when do you name it PMS?[…] Only when you really notice it in your daily life, that it gets in your way.’ P17, female, 38yrs*

For a single GP, it was clear that PMS could take forms as in Premenstrual Dysphoric Disorder if the PMS-symptoms were really intense and had a detrimental effect on the woman´s environment.

*‘I met someone who rebuffed people during that one week, which was not easy to retrieve within three weeks. I saw relationships broken by PMS. I think it requires treatment, so in that sense … an illness. […]I think it´s pathology.’ P23, male, 73yrs*

### A symptomatic treatment approach

Almost all GPs first opted for a symptomatic treatment approach focussing on the symptoms presented and explaining the course of the hormonal cycle. In addition, they advised the women to look for a lifestyle fitting their personal context and for more time and attention for themselves.

*‘Well, […] it's a burden, how much space do you allow yourself, and how much do you want to claim, if you don’t feel well around that period […]: ask for this space!’ P11, male, 65yrs*

Most GPs preferred a hormonal approach as a next step. In shared decision making, they usually prescribed oral contraceptives – cyclically or continuously – or hormone-releasing IUD, possibly assuming that the reduction of hormonal changes would also prevent the occurrence of premenstrual symptoms.

*‘It depends on the wish of the woman, somewhat, and the course of the consultation […] If women want to be treated […], then I think about treatment. But most women reject it, run away from it.’ (Hormones) P8, female, 46yrs*

To support coping strategies and lifestyle, most GPs thought positively about psychological support. They provided this support preferably by themselves or otherwise by their mental health nurse. A minority referred to a psychologist.

*‘When I think: this woman is overburdened, and I suspect aspects of PMS, then I offer support from our mental health nurse.’ P18, female, 48yrs*

Some GPs did not favour a psychological approach because they doubted its benefits. They considered PMS as a hormonal dysfunctional condition that also should be treated as such.

*‘When you think of a physical cause, - that it's in the hormones – and you come up with cognitive behavioural therapy […], it would be one of my last choices. When medical treatment doesn’t work you could add this option.’ P3, female, 36yrs*

Opinions about prescribing antidepressants were explicitly negative. Many GPs never prescribed them because of the side-effects, insufficient experience or because they were generally careful with prescribing antidepressants.

*‘I don’t prefer antidepressants. […]. Since PMS is hormonal … and if you feel fine the other weeks of the cycle, you take the antidepressants during those weeks for nothing.’ P20, male, 65yrs*

As final option many GPs were willingly prescribing them at a later stage, only if really necessary.

*‘Well usually, […]. I won´t. […] If complaints keep disrupting, or patients have a history of psychological complaints, I discuss treatment with an antidepressant, depending on their history.’ P25, female, 45yrs*

## Discussion

### Main findings

This qualitative study sought to investigate the perspective of Dutch GPs towards aetiology, the diagnostic process and subsequent management of PMS.

Although there is ongoing research and debate on its aetiology in the PMS literature, this study does not demonstrate that this is a matter of debate among the 27 interviewed Dutch GPs. The vast majority of the GPs considered PMS to be an intrinsic physiological phenomenon caused by cyclical hormonal changes. They rarely used a symptom diary to confirm the diagnosis. Only some GPs considered PMS to be an illness, in the case of irreversible impairments disrupting the woman's daily life. Others pointed out a correlation between PMS, medically unexplained symptoms, abnormalities in serotonin activity, or a genetic predisposition, but these hypotheses did not seem to affect their proposed PMS policy. Most participants stand for a symptomatic treatment approach, explaining the cyclical nature of the menstrual cycle and offering lifestyle advice fitting to the context of the woman. Using a shared decision-making approach hormonal therapy was advised, usually oral contraceptives or a hormone-releasing IUD. Psychological support was a third step of the policy, given by themselves, or by referral to the mental health nurse, if available in the GP practice, or a psychologist. GPs preferred not to prescribe antidepressants.

#### Comparison with the literature

##### Aetiology of PMS

About the aetiology, we found that GPs merely assumed PMS to be a physiological phenomenon. Why some women get symptoms and others do not is unknown. This finding does not correspond with the current views about PMS/PMDD. Although PMS/PMDD’s aetiology is still not fully understood, a currently widely accepted assumption is that underlying pathophysiology is an abnormal sensitivity to normal changes of ovarian hormones [10,11]. This hormone sensitivity has been associated with genetic and biographic factors like history of trauma [9,10,21,22].

##### Diagnosing PMS

Our finding that most GPs diagnosed PMS/PMDD based on history of the patient is in line with studies indicating that about 90% of clinicians who treat PMDD rely on patients’ retrospective self-report to make the diagnosis [22–24]. However, studies comparing retrospective and prospective PMS/PMDD symptoms have found a remarkable bias towards false positive reports in retrospective self-report measures of premenstrual changes in affect [4,16,25]. Other studies have found that these measures are strongly influenced by clinicians’ beliefs and opinions about PMS [22,23]. The diagnostic process of PMS and PMDD requires a prospective method of monitoring with validated instruments. The cyclicity of symptoms should be determined with a prospectively maintained symptom diary, for at least two consecutive menstrual cycles [26]. In this way, a woman with few pronounced complaints is more likely to receive the correct diagnosis.

##### Impact of PMS on daily life

Our finding that GPs consider PMS as an illness only in case of impairment if PMS disrupts women’s daily life does not fit the diagnosis of PMDD. To meet the DSM-5 diagnosis PMDD, a premenstrual increase in five premenstrual symptoms is necessarily combined with clinically significant distress or interference with work, school, usual activities or relationships with others. In diagnosing PMS per DSM-5, having ´impairment´ is optional. This allows the so-called silent sufferers with distress but without impairment to receive a correct diagnosis as well.

##### Symptomatic treatment approach

In daily practice, GPs strive to clarify patients' complaints in short consultations and propose a suitable approach to their problems in shared decision-making. This fits in with patient-centred care which general practice currently advocates. If desired, and to reduce hormonal changes, the GP can in shared decision-making advise on hormonal contraception. However, there is no conclusive evidence for a long-term effect on PMS complaints [27–29].

GPs did not consider Cognitive Behavioural Therapy (CBT) as a treatment option. There is growing evidence that CBT can contribute to reducing symptoms in women with PMS [30,31].

GPs’ opinions about prescribing antidepressants were explicitly negative; SSRIs are not indicated as first-choice therapy for women with PMS. SSRIs are suitable for the severe forms of PMDD due to their immediate effect, with low or even imtermittent dosage. Nevertheless, SSRIs currently are recommended as gold standard treatment in clinical practice [29,32,33].

##### Strength and limitations

The interviewed GPs constituted a wide variety of gender, age, experience, and practical circumstances. GPs from cultural minorities, however, were lacking. In addition, voluntary participation may have caused selection bias when participants with little interest in PMS are under-represented. Notwithstanding that, we deem PMS views from our participants a true reflection of the commonly applied approach. As qualitative studies by nature are not generalisable to a broader population, this also applies to our study on women with premenstrual syndrome.

##### Implications for practice

We have to develop a reliable and valid diagnostic procedure in primary care for PMS/PMDD. We highly recommend a training and awareness project aimed at increasing knowledge and adequately diagnosing PMS. GPs must acknowledge that for the diagnosis of PMDD, in addition to severe symptoms, the presence of impairment is not required. GPs have to take women’s complaints seriously, regardless of the level of impairment. We advocate the use of a questionnaire and daily symptom monitoring in diagnosing PMS. This will improve the satisfaction of both GPs and women with PMS, as well as the sense of being understood in women with PMS. Highlighting the specific benefits of an SSRI in women with severe premenstrual complaints as PMDD is explicitly part of the training. To provide more evidence for optimal management of PMS, we advocate a study comparing cognitive behavioural therapy with hormonal contraception for women with premenstrual syndrome. The outcome might function as a basis for education and a future guideline for primary care.

In conclusion, most interviewed GPs considered physiological hormonal changes and personal sensitivity important aetiological factors for PMS. Without using a symptom diary for measuring the distress and impairment in their daily lives, they opted for a symptomatic approach to the complaints. They prefer hormonal contraception above SSRIs.

## References

[1] Van der Leij F, Schultz WC, van de Wiel H, et al. [The premenstrual syndrome]. Ned Tijdschr Geneeskd. 2010;154:A1341. Dutch. 20719012

[2] The American College of Obstetricians and Gynecologists. Premenstrual syndrome (PMS). 2020. [cited 2020 December 17]. Available from: https://www.acog.org/clinical/clinical-guidance/committee-opinion/articles/2017/07/mental-health-disorders-in-adolescents

[3] Federation Medical Specialists. 2020. [cited 2020 December 4]. Available from: https://www.richtlijnendatabase.nl/richtlijn/premenstrueel_syndroom/premenstrueel_syndroom_-_startpagina.html. [in Dutch].

[4] Yonkers KA, O'Brien PMS, Eriksson E. Premenstrual syndrome. Lancet. 2008;371(9619):1200–1210. 18395582 10.1016/S0140-6736(08)60527-9 PMC3118460

[5] Kolthof F, Labots-Vogelesang M, Lagro-Janssen T. [The premenstrual syndrome. Prevalence and characteristics in patients in a general practice]. Huisarts Wet. 2005;48:109–112. Dutch.

[6] Potter J, Bouyer J, Trussell J, et al. Premenstrual syndrome prevalence and fluctuation over time: results from a French population-based survey. J Womens Health (Larchmt). 2009;18(1):31–39. 19105683 10.1089/jwh.2008.0932 PMC3196060

[7] Wittchen H-U, Becker E, Lieb R, et al. Prevalence, incidence and stability of premenstrual dysphoric disorder in the community. Psychol Med. 2002;32(1):119–132. 11883723 10.1017/s0033291701004925

[8] Direkvand-Moghadam AK, Sayehmiri K, Delpisheh A, et al. Epidemiology of premenstrual syndrome (PMS)-A systematic review and meta-analysis study. J Clin Diagn Res. 2014;8:106–109. 24701496 10.7860/JCDR/2014/8024.4021 PMC3972521

[9] Hantsoo L, Epperson CN. Allopregnanolone in premenstrual dysphoric disorder (PMDD): Evidence for dysregulated sensitivity to GABA-A receptor modulating neuroactive steroids across the menstrual cycle. Neurobiol Stress. 2020;12:100213. 32435664 10.1016/j.ynstr.2020.100213 PMC7231988

[10] Schmidt PJ, Martinez PE, Nieman LK, et al. Exposure to a change in ovarian steroid levels but not continuous stable levels triggers PMDD symptoms following ovarian suppression. AJP. 2017;174(10):980–989. 10.1176/appi.ajp.2017.16101113 PMC5624833 28427285

[11] Liu Q, Wang Y, van Heck CH, et al. Stress reactivity and emotion in premenstrual syndrome. Neuropsychiatr Dis Treat. 2017;13:1597–1602. 28670129 10.2147/NDT.S132001 PMC5481285

[12] Rapkin AJ, Winer SA. Premenstrual syndrome and premenstrual dysphoric disorder: quality of life and burden of illness. Expert Rev Pharmacoecon Outcomes Res. 2009;9(2):157–170. 19402804 10.1586/erp.09.14

[13] Ussher JM, Perz J. PMS as a gendered illness linked to the construction and relational experience of hetero-femininity. Sex Roles. 2013;68(1-2):132–150.

[14] Eriksson O, Wall A, Olsson U, et al. Women with premenstrual dysphoria lack the seemingly normal premenstrual right-sided relative dominance of 5-HTP-derived serotonergic activity in the dorsolateral prefrontal cortices – A possible cause of disabling mood symptoms. PLoS One. 2016;11(9):e0159538. 27617751 10.1371/journal.pone.0159538 PMC5019404

[15] Gillings MR. Were there evolutionary advantages to premenstrual syndrome? Evol Appl. 2014;7(8):897–904. 25469168 10.1111/eva.12190 PMC4211719

[16] Nevatte T, O'Brien PM, Bäckström T, et al.; Consensus Group of the International Society for Premenstrual Disorders. ISPMD consensus on the management of premenstrual disorders. Arch Womens Ment Health. 2013;16(4):279–291. 23624686 10.1007/s00737-013-0346-y PMC3955202

[17] Ward K, Gott M, Hoare K. Participants' views of telephone interviews within a grounded theory study. J Adv Nurs. 2015;71(12):2775–2785. 26256835 10.1111/jan.12748

[18] ATLAS.ti: the qualitative data analysis & research software. Available from: https://atlasti.com

[19] Malterud K. Systematic text condensation: a strategy for qualitative analysis. Scand J Public Health. 2012;40(8):795–805. 23221918 10.1177/1403494812465030

[20] Tong A, Sainsbury P, Craig J. Consolidated criteria for reporting qualitative research (COREQ): a 32-item checklist for interviews and focus groups. Int J Qual Health Care. 2007;19(6):349–357. 17872937 10.1093/intqhc/mzm042

[21] Ussher JM, Perz J. PMS as a process of negotiation: women's experience and management of premenstrual distress. Psychol Health. 2013;28(8):909–927. 23383644 10.1080/08870446.2013.765004

[22] Cohen LS, Miner CW, Brown EW, et al. Premenstrual daily fluoxetine for premenstrual dysphoric disorder: a placebo-controlled, clinical trial using computerized diaries. Obstet Gynecol. 2002;100(3):435–444. 12220761 10.1016/s0029-7844(02)02166-x

[23] Eisenlohr-Moul TA, Rubinow DR, Schiller CE, et al. Histories of abuse predict stronger within-person covariation of ovarian steroids and mood symptoms in women with menstrually related mood disorder. Psychoneuroendocrinology. 2016;67:142–152. 26896670 10.1016/j.psyneuen.2016.01.026 PMC4811338

[24] Neill Epperson CN, Hantsoo LV. Making strides to simplify diagnosis of premenstrual dysphoric disorder. Am J Psychiatry. 2017;174(1):6–7. 28041003 10.1176/appi.ajp.2016.16101144 PMC5291290

[25] Craner JR, Sigmon ST, Martinson AA, et al. Premenstrual disorders and rumination. J Clin Psychol. 2014;70(1):32–47. 23798035 10.1002/jclp.22007

[26] Bosman RC, Albers CJ, de Jong J, et al. No menstrual cyclicity in mood and interpersonal behaviour in nine women with self-reported premenstrual syndrome. Psychopathology. 2018;51(4):290–294. 29874668 10.1159/000489268 PMC6492812

[27] Lete I, Häusler G, Pintiaux A, et al. The inconvenience due to women's monthly bleeding (ISY) survey: a study of premenstrual symptoms among 5728 women in Europe. Eur J Contracept Reprod Health Care. 2017;22(5):354–359. 29157023 10.1080/13625187.2017.1400001

[28] Leminen H, Heliovaara-Peippo S, Halmesmaki K, et al. The effect of hysterectomy or levonorgestrel-releasing intrauterine system on premenstrual symptoms in women treated for menorrhagia: secondary analysis of a randomized controlled trial. Acta Obstet Gynecol Scand. 2012;91(3):318–325. 22168810 10.1111/j.1600-0412.2011.01340.x

[29] Yonkers KA, Cameron B, Gueorguieva R, et al. The Influence of cyclic hormonal contraception on expression of premenstrual syndrome. J Womens Health (Larchmt). 2017;26(4):321–328. 27854559 10.1089/jwh.2016.5941 PMC5397197

[30] Ismaili E, Walsh S, O'Brien PMS, et al.; Consensus Group of the International Society for Premenstrual Disorders. Fourth consensus of the International Society for Premenstrual Disorders (ISPMD): auditable standards for diagnosis and management of premenstrual disorder. Arch Womens Ment Health. 2016;19(6):953–958. 27378473 10.1007/s00737-016-0631-7

[31] Lustyk MKB, Gerrish WG, Shaver S, et al. Cognitive-behavioral therapy for premenstrual syndrome and premenstrual dysphoric disorder: a systematic review. Arch Womens Ment Health. 2009;12(2):85–96. 19247573 10.1007/s00737-009-0052-y

[32] Sepede G, Sarchione F, Matarazzo I, et al. Premenstrual dysphoric disorder without comorbid psychiatric conditions: a systematic review of therapeutic options. Clin Neuropharmacol. 2016;39(5):241–261. 27454391 10.1097/WNF.0000000000000173

[33] Lovick T. SSRIs and the female brain-potential for utilizing steroid-stimulating properties to treat menstrual cycle-linked dysphorias. J Psychopharmacol. 2013;27(12):1180–1185. 23704364 10.1177/0269881113490327

